# Differential Effects of Zooplankton on Sunlight Inactivation of Viruses

**DOI:** 10.1021/acs.est.6c03486

**Published:** 2026-05-26

**Authors:** Martha I. Verbel-Olarte, Tamar Kohn, Niveen S. Ismail

**Affiliations:** † Laboratory of Environmental Virology, School of Architecture, Civil and Environmental Engineering (ENAC), École Polytechnique Fédérale de Lausanne (EPFL), 1015 Lausanne, Switzerland; ‡ Picker Engineering Program, 6089 Smith College, Northampton, Massachusetts 01063, United States

**Keywords:** Viral inactivation, zooplankton grazing, solar disinfection, water treatment, microbial water quality

## Abstract

Interactions between viruses and filter-feeding zooplankton can alter viral persistence in surface waters, with direct implications for water quality and public health risk. However, data on virus-zooplankton interactions and the environmental factors that influence them are still limited. This study evaluated the impact of filter feeding, in the dark and under simulated sunlight, on a bacteriophage (MS2) and a human virus (echovirus11; E11) in the presence of a ciliate (*Tetrahymena pyriformis*) and rotifer (*Brachionus calyciflorus*). Dark experiments established organism-dependent baseline removal for each virus, and rotifers showed greater removal of both viruses in comparison to ciliates. Under simulated sunlight, in contrast, experiments with ciliates resulted in greater virus removal compared to experiments with rotifers over a similar timespan (4.2 vs 2.7 log MS2 in 53–58 h; 3.5 vs 3.0 log E11 in 24–25 h). Analysis of decay rate constants reveals species-specific shifts in virus removal between dark and light that, depending on viral type and zooplankton species, either accelerate viral attenuation or protect viruses and prolong infectivity. *T. pyriformis* increases removal under sunlight relative to dark conditions and acts synergistically with sunlight inactivation, whereas rotifers impede sunlight inactivation. These data address important knowledge gaps on how dark biotic processes can modulate sunlight-mediated inactivation. The reported decay constants can help inform predictions of virus environmental fate with implications for natural and engineered treatment systems that rely on sunlight disinfection to inactivate viruses.

## Graphical abstract

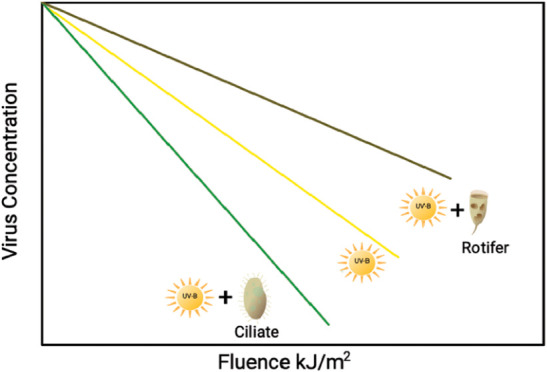

## 1 Introduction

Filter-feeding zooplankton regulate water quality by removing and ingesting suspended organic particles across a broad size range, from bacteria to algae. While their contribution to water quality improvement is well documented,^1^ the interactions between zooplankton and microbial pollutants, particularly viruses, are complex and variable.^2–7^ Zooplankton can reduce viral loads through predation and inactivate viruses during digestion, yet they can also harbor viruses^4,5,8–13^ and excrete infectious viruses in feces,^14,15^ potentially serving as vectors. Viral inactivation is strongly dependent on environmental conditions,^16^ and zooplankton-virus interactions vary with both zooplankton species and virus type,^7,9,17–19^ but available data are insufficient to predict when grazing will contribute to viral decay or prolong infectivity. Understanding zooplankton-virus interactions and their influence on other environmental processes that modulate viral fate is essential for effective water quality management.

Interactions between zooplankton and viruses have direct consequences for human health and water safety. Enteric viruses in water bodies can cause illnesses such as gastroenteritis and respiratory infections,^20,21^ and elevated microbial pollutant concentrations can prompt recreational water closures with substantial economic consequences.^22^ To inform water quality management, research has characterized and modeled viral inactivation driven by abiotic factors, such as temperature and pH, with sunlight recognized as a major driver of viral inactivation.^16,23–28^ Environmental conditions and water chemistry, which modulate virus aggregation and surface adhesion, modify the efficacy of sunlight inactivation of viruses.^23,29,30^ The impact of filter-feeding zooplankton on sunlight inactivation, however, remains inadequately characterized.

Most prior studies addressed zooplankton effects on chemical and UV254 disinfection of bacteria,^31–33^ or examined amoebae that use a predatory engulfing strategy rather than filter-feeding.^4,9^ Numerous studies have demonstrated protective effects for bacteria within protists. For example, *Tetrahymena sp.* can protect coliform bacteria from chlorination,^34^ and *Campylobacter* from virudine disinfectant.^35^ One study observed protection of Φ174 and MS2 from UV by several *Tetrahymena* species.^10^ For rotifers, studies have shown that they can harbor *Vibrio*, but protection effects specific to rotifers and bacteria or viruses are not well characterized.^36^ To date, only one study has shown protection of MS2 from sunlight by the brackish-water rotifer, *Brachionus plicatilis*.^37^

In this study, we evaluate the effect of two filter-feeding zooplankton species, the ciliated protist *Tetrahymena pyriformis* and the rotifer *Brachionus calyciflorus*, on the decay of echovirus 11 (E11) and bacteriophage MS2 in the presence and absence of sunlight. E11, an enterovirus associated with a broad spectrum of human illnesses,^38^ is commonly detected in environmental waters and wastewater,^39^ and has been shown to be removed by filter-feeding zooplankton.^7,12^ MS2 is widely used as a surrogate for human enteric viruses^18^ and is considered a conservative indicator of sunlight inactivation of these viruses. Its removal has also been investigated in coincubation studies with filter-feeding zooplankton.^7,18^ Studying these two viruses enables comparison with prior work while providing new insights into zooplankton-mediated effects on their environmental persistence.

The two zooplankton species were chosen for their relevance to both natural freshwater environments and engineered treatment systems, as well as their extensive use as model organisms.^40,41^ *T. pyriformis* has been widely adopted as a model species, yet prior research has reported substantial variability in viral uptake, even when examining the same virus across different studies.^7,12,13,42^ In contrast, viral interactions with *B. calyciflorus* remain relatively understudied, providing an opportunity to extend understanding beyond the limited data available for the brackish-water rotifer *B. plicatilis*. Given the variability in findings on viral-zooplankton dynamics and the scarcity of research on their role in sunlight inactivation of viruses, this study aims to quantify how dark and sunlight inactivation rates vary among viruses and zooplankton species within a single system.

## 2 Materials and Methods

### 2.1 *T. pyriformis* Culture and Preparation

Cultures of *T. pyriformis* ciliates (Culture Collection of Algae and Protozoa 1630/1W) were maintained axenically in proteose peptone yeast extract (PPYE) medium at 24 °C in 150 cm^2^ cell culture flasks (TTP, Milian). PPYE was prepared by dissolving 20.0 g of proteose peptone (VWR) and 2.5 g of yeast extract (Thermo Scientific) in 1 L of MilliQ water. The solution was autoclaved and stored at 4 °C. Subcultures were axenically prepared weekly in a laminar flow hood by spiking 1 mL of the previous culture into 39 mL of fresh PPYE (total volume of 40 mL). One day prior to the experiments, ciliates were washed according to previously published protocols.^3,7,12^ Briefly, 120 mL of *T. pyriformis* in PPYE were centrifuged at 400*g* for 10 min to form a pellet. The supernatant was removed, and 10 mL of sterile phosphate-buffered saline (PBS) (Table S1) was added. The pellet was gently resuspended and centrifuged at 400*g* for 6 min. The resuspension procedure was repeated twice with PBS, and the final pellet was suspended in 50 mL of filter-sterilized, moderately hard synthetic freshwater (MHSFW) (Table S2).^43^ The *T. pyriformis* suspension was left at room temperature for approximately 20 h before use. *T. pyriformis* were enumerated using an automated cell counter in brightfield mode (DeNovix, CellDrop).

### 2.2 *B. calyciflorus* Culture and Preparation

*B. calyciflorus* resting cysts (Florida Aquafarms) were hatched in 30 mL of sterile MHSFW and incubated at 25 °C under low light intensity (17 μmol photons m^–2^ s^–1^) in an incubator (AlgaeTron AG 230) on a 16h:8h light: dark cycle. Upon hatching, rotifers (20–50 individuals) were transferred into a 1 L glass bottle containing 900 mL of MHSFW with high aeration and fed daily with 25 mL of the algae *Chlorella vulgaris* (SI text for culture details and Table S3). Cultures were refreshed weekly; one-half of the culture was discarded, and aggregates of algae and dead rotifers were removed by sieving through a 125 μm sieve. The collected filtrate from the 125 μm was subsequently sieved through a 50 μm sieve to collect the rotifers. The rotifers were removed from the sieve and resuspended in 900 mL of MHSFW. Two hours prior to each experiment, rotifers were concentrated using a 50 μm sieve and resuspended to the desired experimental density. Rotifers were enumerated using a gridded Sedgewick Rafter (Wildco) using an inverted light microscope (Olympus CK X41).

### 2.3 Virus Propagation and Preparation

Echovirus 11 (E11) (Gregory strain, ATCC VR37) was propagated on Buffalo green monkey kidney (BGMK) cells (kindly provided by the Spiez Laboratory, Switzerland) using a previously published protocol.^44^ E11 stock solutions were purified using 100 kDa centrifugal filters (Centricon), then stored at −20 °C and thawed immediately before use. Infectious E11 concentrations were determined by the most probable number (MPN) assay using serial 10-fold dilutions (20 μL inoculum) on 95% confluent BMGK cells incubated for 5 days following previously published methods.^44^ E11 concentrations were reported as most probable number or cytopathic units per milliliter (MPNCU mL^–1^). RStudio software (Version 2023.06.1 + 524) was used to calculate the E11 MPNCU mL^–1^ from the infectivity assay. The limit of quantification (LOQ) of the MPN assay is 30 MPNCU mL^–1^, corresponding to the lowest E11 concentration with a 95% probability of detecting at least one well with a positive cytopathic effect under the experimental conditions.

MS2 (DSM 13767) bacteriophage was replicated in *Escherichia coli* (DSM 5695) host. Phage purification and propagation followed previously published protocols, with slight modifications.^45^ Briefly, bacterial cell debris was removed by centrifugation at 4000*g* for 15 min, followed by vacuum filtration through a 0.45 μm PVDF membrane (Stericup) under sterile conditions. The MS2 was further purified using 100 kDa centrifugal filters by transferring 65 mL of phage solution to the filters and centrifuging at 3000*g* for 30 min. An equal volume of PBS (65 mL) was added, followed by centrifugation. The purified viral stock solution was then eluted at 3000*g* for 2 min and stored at −20 °C. Infectious MS2 (100 μL samples) was enumerated by the double-layer agar method, and concentrations are reported as plaque-forming units per mL (PFU mL^–1^).^37,46^ The LOQ for MS2 is 300 PFU mL^–1^.

### 2.4 Virus and Zooplankton Co-incubation Experiments

#### 2.4.1 Sunlight Exposure Experiments

Simulated sunlight exposure experiments were conducted using a solar simulator (Sun 2000; Abet Technologies) equipped with a 1000-W Xenon lamp, an Air Mass (AM) 1.5 filter, and a 2-mm thick atmospheric edge (AE) filter. The lamp was operated at 30 A to preserve healthy zooplankton populations while still allowing measurable sunlight inactivation kinetics. The filters were used to mimic the solar radiation spectrum, resulting in spectral irradiance across the entire solar spectrum.^47^ The irradiance spectrum was measured at four locations below the simulator using a spectroradiometer (ILT 900-R; International Light Technologies, Peabody, MA) (Figure S1). The average UVB irradiance, calculated over the wavelength range from 280 to 315 nm, was 0.17 ± 0.005 W m^–2^. We focus our study on the 280–315 nm wavelength range since endogenous inactivation is driven primarily by UVB.^23,48^ Although UVA/visible light can contribute to exogenous inactivation in the presence of sensitizer and reactive oxygen species (ROS), UVB can still dominate exogenous inactivation as well, due to higher photon energy and stronger absorption of sensitizers at shorter wavelengths.^49–51^

Six experimental beakers (*n* = 6 per virus and zooplankton combination) contained zooplankton (*T. pyriformis* or *B. calyciflorus*) in 150 mL MHSFW (water depth, *z* = 3.5 cm) coincubated with virus to achieve initial concentrations of 10^6^ PFU mL^–1^ for MS2 and 10^5^ MPNCU mL^–1^ for E11. The 400 mL glass beakers were painted black to prevent sunlight from entering through the sides. Beakers were placed in a recirculating water bath to maintain a temperature of 24 °C (Figure S2). Constant aeration was provided using sterilized aquarium air stones (Tetra) to maintain mixing and sufficient dissolved oxygen levels (Figure S2), and beakers were covered with plastic film to minimize evaporation and aerosolization of the viruses. The plastic film did not shield light in the solar spectrum. The average initial experimental *T. pyriformis* density was 10^5^ ciliates mL^–1^ and the average initial density for *B. calyciflorus*, was 130 rotifers mL^–1^. Zooplankton viability and densities were checked at the start, midpoint, and end of each experiment. Concentrations varied by less than 10% for rotifers (Figure S3) and less than 15% for ciliates (Figure S4) over each experiment (See SI Text and Table S4 for further analysis). MS2 light exposures lasted 53–58 h, and the E11 exposures lasted 24–25 h. These different exposure durations were selected based on expected sunlight inactivation rates, with MS2 serving as a conservative indicator of sunlight inactivation of enteric viruses due to its slower decay rate.^52^ Samples were collected periodically (1 mL), filtered through a 0.22 μm sterile syringe filter (FILTER- BIO) to remove zooplankton, and infectious virus concentrations were enumerated. Unfiltered aliquots (2 mL) taken at the start and end of the experiments were utilized for UV–vis absorbance measurements (see below), zooplankton enumeration, and assessment of zooplankton viability through visual inspection using an inverted light microscope. Virus-only controls (*n* = 2 beakers) were run in sterile MHSFW in parallel to quantify viral sunlight inactivation in the absence of zooplankton.

#### 2.4.2 Dark Experiments

Dark experiments with zooplankton (*n* = 6) and controls without zooplankton (*n* = 3) followed the same protocol as the sunlight-exposure experiments, except that the beaker tops were covered with aluminum foil to exclude light. Dark experiments quantified dark biotic removal of viruses and baseline dark decay due to abiotic losses. Incubation durations were chosen based on the duration of the sunlight inactivation experiments. For MS2, dark experiments ran for 55 h, and for E11, exposure ran for 30 h.

### 2.5 Data Analysis

The irradiance values were corrected for light absorbance by solutions containing rotifers and ciliates using a correction (screening) factor (eq 1).^23,53^ The absorbance spectrum (α_s_) of each solution was obtained using a UV–vis with an integrating sphere (Shimadzu, model UV 2450–2550) to account for light scattering by the solution (Figure S5).

1 $$\left\langle I_{\mathrm{avg}}\left(z,\lambda \right)\right\rangle =I_{0}\left(0,\lambda \right)\left(\frac{1-10^{-\alpha_{s}\left(\lambda \right)z}}{2.303\alpha_{s}\left(\lambda \right)z}\right)$$

where ⟨*I*_avg_ (*z*,λ)⟩ in W cm^–2^ nm^–1^ is the spectral irradiance averaged over the depth *z* of the experimental solution in each beaker in cm, *I*_0_ (0,λ) is the incident spectral irradiance in W cm^–2^ nm^–1^, and α_s_ (λ) is the absorbance spectrum of the experimental solutions in cm^–1^.

Rate constants were calculated with respect to time (*k*, hr^–1^) and fluence (κ, m^2^ kJ^–1^) for pooled samples across all experimental replicates using a first-order decay model

2 $$C_{t}=C_{o}e^{-\mathrm{kt}}$$

3 $$C_{t}=C_{o}e^{-\kappa F_{\mathrm{avg}}}$$

where *C*_o_ is the virus concentration at *t* = 0 h in PFU mL^–1^ for MS2 or MPNCU mL^–1^for E11, *C*_*t*_ is the virus concentration at a given time point, *t* is time in hours, and *F*_avg_ is the UVB fluence averaged over the depth *z* of the experimental solution in each beaker in kJ m^–2^ calculated as

4 $$F_{\mathrm{avg}}=\sum_{\lambda =280}^{315}\left[I_{\mathrm{avg}}\left(z,\lambda \right)\right]t$$

We used a constant solution depth of *z* = 3.5 cm for all *F*_avg_ calculations. Only 11 mL of liquid volume was removed cumulatively by the end of the last sampling point. Considering a 20 mL volume loss as a worst-case scenario, this would lead to a 0.2 cm change in depth and an insignificant change in *F*_avg_. *F*_avg_ is referred to as fluence throughout the manuscript.

All statistical analyses were completed using SPSS (v28, IBM). All viral concentration data were log-transformed before statistical analysis. Results were considered significant for *p* < 0.05. The mean ± standard deviation or propagated error values are presented for experimental data. Both standard deviation and propagated error are referred to as SD in the manuscript. The standard error (SE) is reported for calculated rate constants (*k*-value and κ-values). Repeated-measures ANOVA and linear mixed models (LMM) were used to test for differences in viral concentration across treatments in the experiments. Spearman’s rank order correlation was used to test for relationships between variables.

## 3 Results and Discussion

### 3.1 Virus Type and Zooplankton Species Impact the Effectiveness of Dark Inactivation

The removal rates of MS2 and E11 were first quantified in the dark, in the presence of *T. pyriformis* and *B. calyciflorus*, with appropriate controls (Figure 1A and B). When MS2 was incubated with *T. pyriformis*, decay was not significantly different from the virus-only control (ANOVA, *p* = 0.180, Figure 1A). After 55 h, treatments with ciliates showed a 0.7 ± 0.1 log removal, while virus-only controls showed a 0.6 ± 0.1 log decay. Previous studies reported higher MS2 removal by *T. pyriformis*: 1.3 log removal in ciliate treatments versus 0.6 log decay in virus-only controls over 48 h in MHSFW,^7^ and about 1.1 log removal with ciliates and 0.5 log decay in virus-only controls after 48 h in a salt solution.^18^ While these values are numerically higher than ours, direct comparison is not feasible due to differences in experimental conditions, including duration and water matrix. Despite these differences, when considering net log removal (treatment minus control) for each reported experiment, the removals are less than 1 log. In contrast, *B. calyciflorus* produced a 1.5 ± 0.01 log removal of MS2 in the dark compared with a 0.6 ± 0.1 log reduction in the virus-only controls after 53 h. Across the full time series, the decline in virus concentration in the rotifer treatments was significantly greater than in the virus-only controls (ANOVA, *p* = 0.032, Figure 1A). Comparative data for rotifers are limited, but *B. plicatilis*, a brackish-water rotifer of the same genus with similar feeding behavior, achieved a net 2 to 3 log removal of MS2 (after accounting for decay in the control) over 96 h in brackish water.^37^

**Figure 1:**
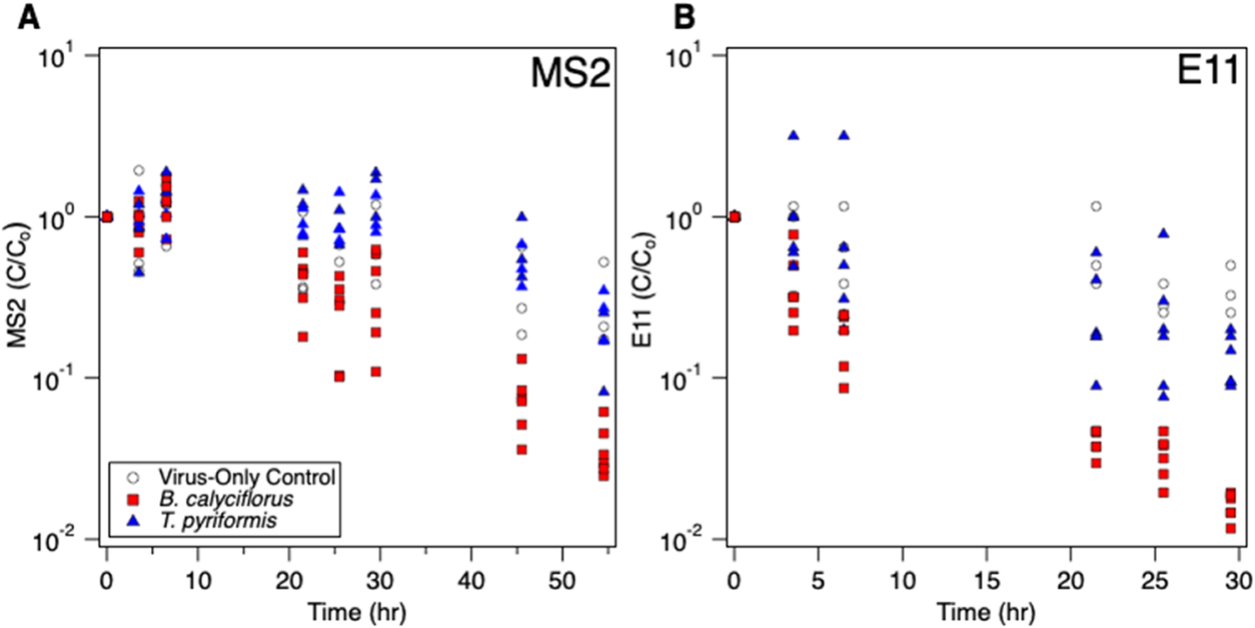
Viral decay in controls without zooplankton and removal kinetics during coincubation with zooplankton in the dark. (A) MS2 and (B) E11. Six replicates were tested with zooplankton, and virus-only controls without zooplankton were tested in triplicate. Note the differing time scales on the x-axes for the two viruses.

Similar findings were obtained for E11. Incubation with *T. pyriformis* did not significantly increase dark removal relative to the control with 0.9 ± 0.1 log removal over 30 h with ciliates and 0.5 ± 0.1 log decay in virus-only controls (ANOVA, *p* = 0.258, Figure 1B). Previous work has shown that E11 removal by *T. pyriformis* can require up to 96 h to achieve 1.5 log removal, with only 0.3 log removal at 48 h.^12^ In contrast, *B. calyciflorus* significantly enhanced E11 removal in the dark, achieving 1.8 ± 0.01 log removal versus a 0.5 ± 0.1 log decay in virus-only controls at approximately 30 h (ANOVA, *p* < 0.001, Figure 1B). Although *B. calyciflorus* removed both MS2 and E11, the MS2-rotifer system required 55 h to reach 1.5 log decline, whereas E11 reached 1.8 log decline within 30 h under the same dark conditions.

The dark experiments established baseline information on virus stability in the water matrix used herein and showed differences in removal as a function of zooplankton species and virus. Previous research has shown variability in viral uptake by *T. pyriformis*,^7,10,18^ but the underlying causes remain unclear. For example, Olive et al. found that uptake is virus-specific and may be linked to virus surface hydrophobicity, but not to virus size.^7^ In our study, E11 and MS2 are similarly sized, yet significant differences in removal were observed (LMM, *p* < 0.001), consistent with the findings of Olive et al.^7^

Mechanistic data on rotifer uptake of viruses are limited beyond MS2 removal by *B. plicatilis*.^37^ Studies of other particle types indicate that *B. calyciflorus* may alter feeding behavior according to particle characteristics, concentration, and size.^54^ Rotifers can ingest particles in the bacterial size range, but their ciliary feeding structures are not optimized to collect particles in the submicron range.^54,55^ However, in our experiments, rotifers did remove viruses in the 27–30 nm range.

Given the zooplankton densities used in this study and published clearance rate ranges, both *T. pyriformis* and *B. calyciflorus* would be expected to have similar theoretical capacities to process the beaker contents. Reported clearance rates for *T. pyriformis* on 1 μm microspheres (surrogates for bacteria) suspended in growth media are 5 × 10^–5^ to 1 × 10^–4^ mL ciliate^–1^ hr^–1^,^56^ which at our density (10^5^ ciliates mL^–1^) corresponds to roughly 130–250 passes of the beaker over 25 h. For E11 specifically, the clearance rate for starved *T. pyriformis* in MHSFW has been estimated at 1 × 10^–7^ mL ciliate^–1^ hr^–1^, corresponding to 0.25 passages over 25 h.^5,7^ For *B. calyciflorus*, reported bacterial clearance rates range from 1 × 10^–4^ to 5 × 10^–2^ mL rotifer^–1^ hr^–1^,^54^ corresponding to 0.33 to 160 passes of the beaker over 25 h using our experimental density of 130 rotifers mL^–1^. Although these published values are not specific to our exact conditions, they suggest that, despite the orders of magnitude difference in numerical density between ciliates (∼10^5^) and rotifers (∼10^2^) in our experiments, both species can have comparable theoretical capacities for water processing because the higher per-individual clearance rates for rotifers can compensate for the lower numerical abundance. However, we observed significantly different removal between *T. pyriformis* and *B. calyciflorus* for both MS2 and E11 (ANOVA, *p* < 0.001), underscoring the biological complexity and the need to further elucidate the mechanisms driving these differences.

### 3.2 Sunlight Inactivation of Viruses is Variably Impacted by Ciliates and Rotifers

Figure 2A and 2B compare MS2 removal by simulated sunlight in the absence and presence of *T. pyriformis*. Time-based analysis (Figure 2A) of decay rates of MS2 with and without *T. pyriformis* revealed a statistically significant difference in residual virus titer after 40 h (LMM, *p* = 0.010). After correcting for absorbance (eq 1, Figure S5), treatments with *T. pyriformis* exhibited a steeper decline than the controls after 8 h (Figure 2B). Virus-only controls in sunlight achieved a total decay of approximately 2.5 log at a fluence of 32 kJ m^–2^, whereas the presence of *T. pyriformis* significantly enhanced MS2 decay to approximately 4.2 log at a fluence of only 17 kJ m^–2^ (LMM, *p* = 0.029) (Figure 2B). Note that the strong absorbance of solutions containing *T. pyriformis* (Figure S5) caused the pronounced reduction in the fluence in *T. pyriformis* experiments compared to the virus-only controls, despite equal experimental times. For *B. calyciflorus* experiments (Figure 2C and D), virus-only controls in sunlight resulted in approximately 2.0 log decay at a fluence of 29 kJ m^–2^ compared with 2.7 log decay at a fluence of 31 kJ m^–2^. Although there was a 0.7 log difference between the control and experimental treatments at the end point, this difference was not statistically significant when considering the entire time series (LMM, *p* = 0.174) or fluence data (LMM, *p* = 0.171).

**Figure 2:**
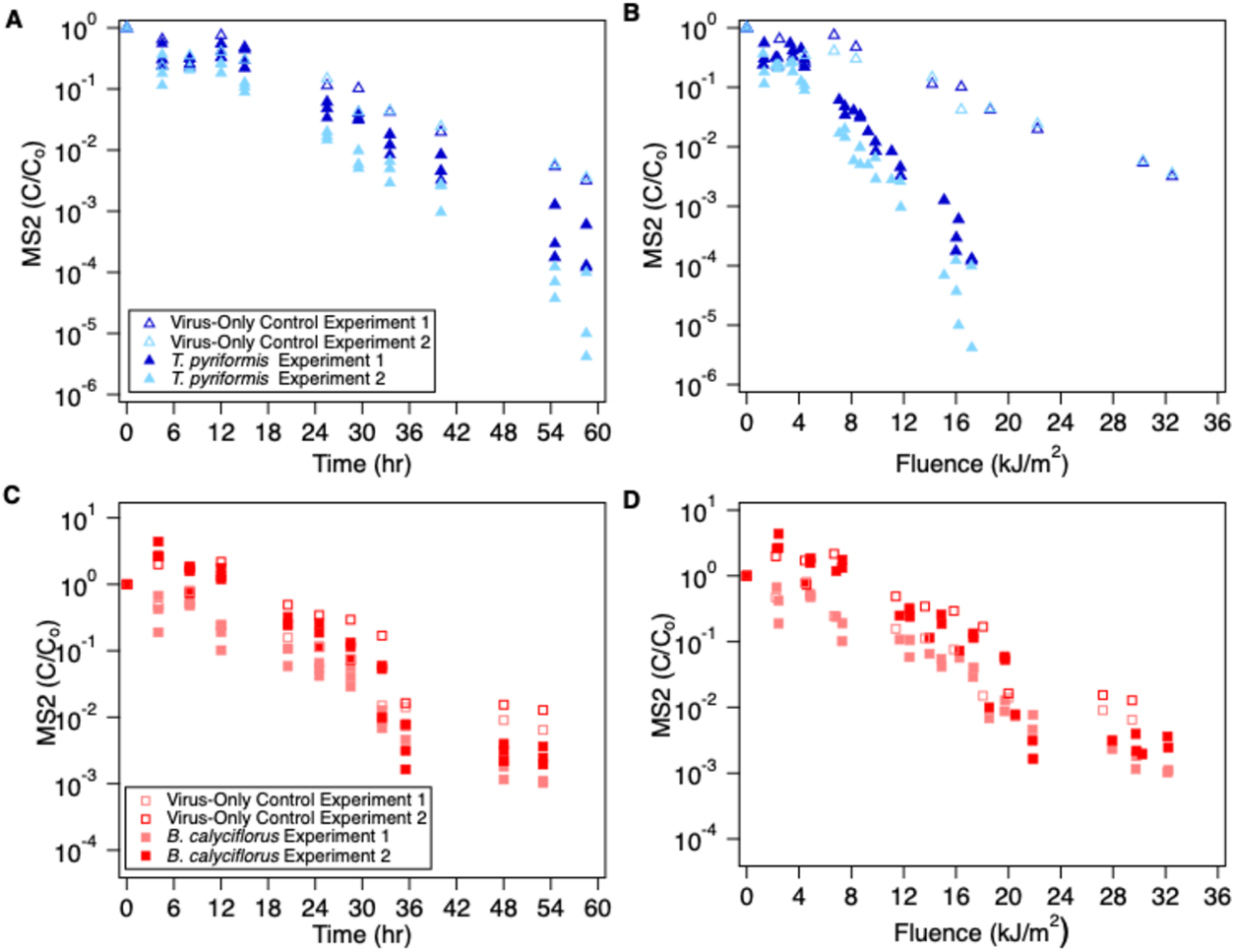
Sunlight inactivation of MS2 in the absence and presence of zooplankton. Panels A and B show the effect of *T. pyriformis* on MS2 concentration as a function of time and fluence, respectively. Panels C and D show the impact of *B. calyciflorus* as a function of time and fluence, respectively. Data from three replicates are presented for each of the zooplankton experiments (*n* = 3). Note the difference in *y*-axis values for *T. pyriformis* (A and B) versus *B. calyciflorus* (C and D).

*T. pyriformis* also significantly enhanced E11 removal under sunlight. An enhancement was observed when E11 decay was analyzed as a function of time (Figure 3A) (LMM, *p* = 0.044), and it was even more pronounced when analyzed as a function of fluence, with a 3.5 log decay at 7.3 kJ m^–2^ fluence in the presence of ciliates, compared with 2.2 log decay at 14.1 kJ m^–2^ fluence in the virus-only controls (LMM, *p* = 0.002) (Figure 3B). In contrast, *B. calyciflorus* significantly protected E11 from sunlight inactivation. This effect, too, was evident both as a function of time (LMM, *p* < 0.001) and fluence (LMM, *p* < 0.001) (Figure 3C and D), with 3.0 log decay at 14.5 kJ m^–2^ fluence with rotifers versus 4.0 log decay at 11.2 kJ m^–2^ fluence for the virus-only controls.

**Figure 3:**
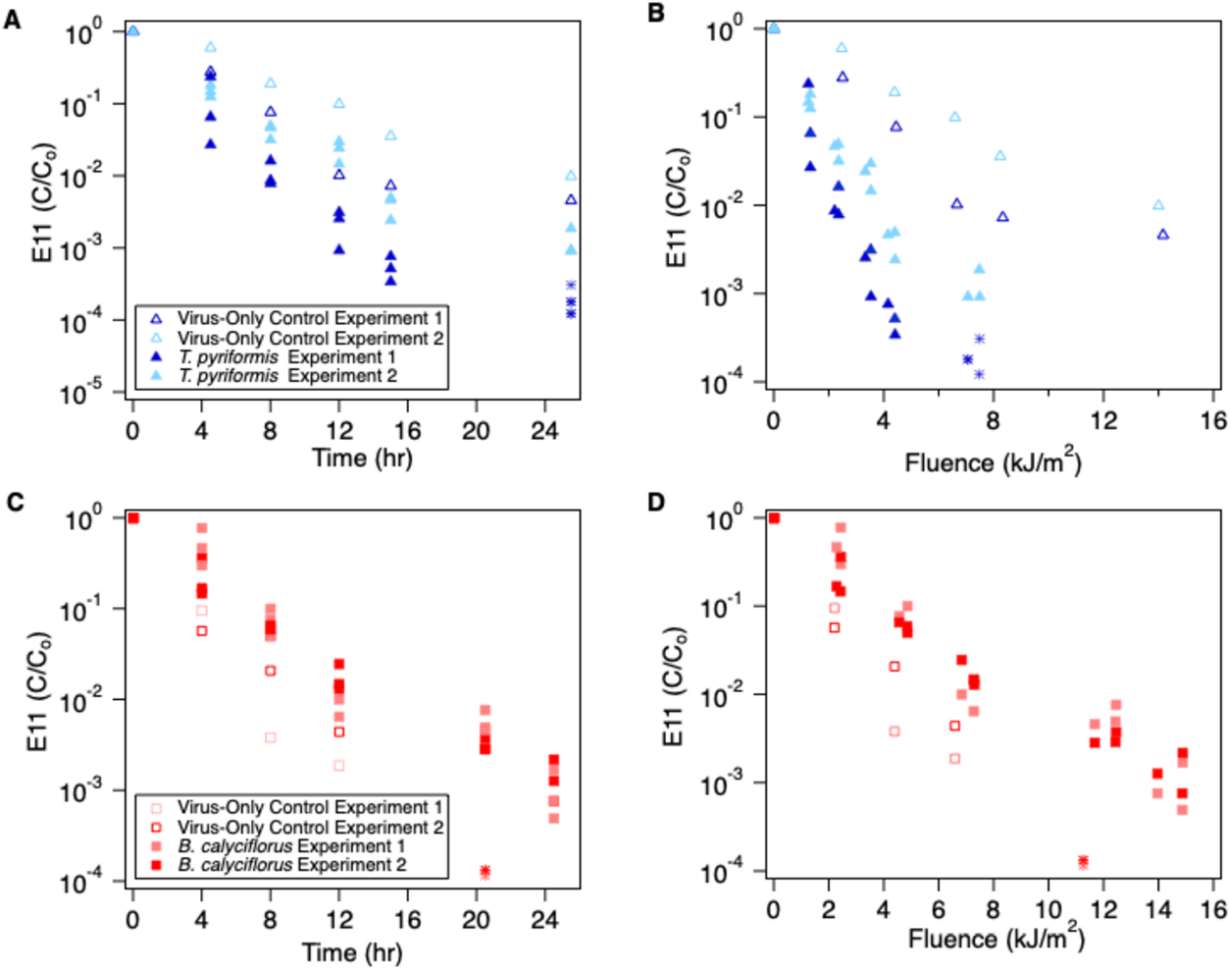
Sunlight inactivation of E11 in the absence and presence of zooplankton. Panels A and B show the effect of *T. pyriformis* on E11 concentration as a function of time and fluence, respectively. Panels C and D show the impact of *B. calyciflorus* as a function of time and fluence, respectively. Data from three replicates are presented for each of the zooplankton experiments (*n* = 3). Star symbols indicate infectious virus concentrations below the limit of quantification.

Virus-only controls for E11 exhibited greater decay for *B. calyciflorus* versus for *T. pyriformis*, possibly due to the sensitivity of E11 to aeration. Vigorous aeration was necessary for *B. calyciflorus* experiments to provide sufficient dissolved oxygen, whereas for *T. pyriformis*, lower aeration was provided to ensure adequate mixing while avoiding cyst formation or organismal death. Because a simple aquarium air pump was used, air flow rates were not quantified. In contrast, aeration sensitivity was not observed for MS2. Virus-specific sensitivity to aeration has been reported previously. For example, Qβ was sensitive to aeration, while MS2 and ΦX174 were not.^57^ Additional controlled experiments could further elucidate the mechanisms underlying viral sensitivity to aeration and clarify other contributing factors, but such investigations are beyond the scope of the present study.

Limited knowledge exists regarding how rotifers and ciliates affect the inactivation of viruses by sunlight. One study reported modest protection (3–8% reduction in inactivation) of ΦX174 and MS2 by several *Tetrahymena* species, including *T. pyriformis*, during a 3-min exposure to UVB light.^10^ Our results contrast with these previous findings, as decay of both E11 and MS2 under sunlight in the UVB range was enhanced in the presence of *T. pyriformis*. Differences in system conditions, including media type and exposure conditions, may explain the conflicting results. For rotifers, protection of MS2 from sunlight by *B. plicatilis* in brackish water has been reported.^37^ In our system, *B. calyciflorus* in MHSFW strongly protected E11, but effects on MS2 were minor and not statistically significant. Several experimental differences could explain these contrasting outcomes: water matrix (brackish versus MHSFW), aeration intensity, and sunlight exposure conditions (variable natural sunlight irradiance versus steady irradiance produced with a solar simulator). In addition, although the rotifers belong to the same genus, species-specific feeding traits and behavior^58^ may influence viral removal. Hence, these differences limit direct comparison between studies and warrant further investigation to identify underlying causes.

### 3.3 Comparison of Inactivation Rate Constants Reveals Synergistic Effect of *T. pyriformis* and Antagonistic Effects of *B. calyciflorus* on Sunlight Inactivation

To further quantify zooplankton impacts on virus inactivation under dark and sunlight conditions, we calculated inactivation rate constants with respect to time (*k*, hr^–1^) and fluence (κ, m^2^ kJ^–1^) (Table 1). Because solution absorbance differed between treatments, fluence-based κ- values are the most appropriate metric for comparing sunlight experiments. Experimental to virus-only control ratios for time-based *k* (dark) and fluence-based κ (light) were calculated to facilitate comparisons of zooplankton effects in dark and light conditions (Table S5). For example, for E11 with *T. pyriformis*, the dark *k*-ratio (experimental/virus-only control) was 1.8 while the light κ-ratio was 3.1, suggesting an approximately 1.7 fold enhancement of removal by *T. pyriformis* in the light. A similar pattern was observed for MS2 with *T. pyriformis*: the dark *k*-ratio was 0.66, but as previously noted, there was no significant difference in dark experimental (with *T. pyriformis*) and dark control (virus-only) time series data. In the light for *T. pyriformis*, the κ-ratio was 3.2 for MS2. Combined, these analyses indicate altered feeding or processing behavior of *T. pyriformis* in sunlight in comparison to the dark. By contrast, *B. calyciflorus* showed greater removal than the virus-only control in the dark for E11 with a *k*-ratio of 3.8, but they produced a fractional κ-ratio of 0.57 in the light, indicating protection of E11 from the disinfection effects of sunlight. While a similar trend in the ratios is also observed for *B. calyciflorus* coincubation with MS2, as previously discussed, differences between the control and experimental time-series and fluence-based data under sunlight were not statistically significant.

**Table 1:** Virus Inactivation Rate Constants Based on Time (*k*, hr^–1^) and Fluence (κ, m^2^ kJ ^–1^) under Light and Dark Conditions, with and without Zooplankton (Virus-Only Control)^a^

Condition	*T. pyriformis* Experimental	Virus-Only *T. pyriformis* Control	*B. calyciflorus* Experimental	Virus-Only *B. calyciflorus* Control
Dark E11	0.068 ± 0.008 h^–1^	0.038 ± 0.005 h^–1^	0.14 ± 0.006 h^–1^	0.038 ± 0.005 h^–1^
Light Ell	0.37 ± 0.02 h^–1^	0.23 ± 0.02 h^–1^	0.29 ± 0.007 h^–1^	0.47 ± 0.02 h^–1^
	1.3 ± 0.06 m^2^ kJ^–1^	0.42 ± 0.03 m^2^ kJ^–1^	0.49 ± 0.01 m^2^ kJ^–1^	0.86 ± 0.04 m^2^ kJ^–1^
Dark MS2	0.016 ± 0.005 h^–1^	0.024 ± 0.002 h^–1^	0.056 ± 0.005 h^–1^	0.024 ± 0.002 h^–1^
Light MS2	0.15 ± 0.004 h^–1^	0.094 ± 0.003 h^–1^	0.12 ± 0.005 h^–1^	0.088 ± 0.006 h^–1^
	0.54 ± 0.01 m^2^ kJ^–1^	0.17 ± 0.006 m^2^ kJ^–1^	0.20 ± 0.008 m^2^ kJ^–1^	0.16 ± 0.01 m^2^ kJ^–1^

a Standard errors are reported for each rate constant.

Additive effects for *T. pyriformis* were assessed by comparing the sum of dark inactivation by *T. pyriformis* (*k*_Dark, *T. pyriformis*_) and sunlight inactivation for the virus-only control (*k*_Light,virus‑onlycontrol_) with the experimental sunlight inactivation with *T. pyriformis* (*k*_Light, *T. pyriformis*_) (Table S6). For both MS2 and E11, the summed time-based rate constants were lower than the observed *k*_Light, *T. pyriformis*_; for MS2, the values were 0.11 versus 0.15, and for E11, 0.30 versus 0.37, indicating synergistic enhancement of inactivation when grazing and sunlight act together. Because time-based inactivation rates do not account for differences in solution absorbance and delivered fluence, these comparisons likely underestimate the synergistic effect.

Differences in decay rate constant ratios between dark and light conditions may reflect behavioral and physiological changes in zooplankton. Phototactic responses differ among zooplankton. *Brachionus spp.* possess an eye spot that senses light, and exhibit phototaxis that varies with light intensity and wavelength.^59^ Rotifers show positive phototaxis at different wavelengths in the visible light spectrum and negative phototaxis in UVA light,^60^ but UVB light has not been examined. Although *T. pyriformis* does not have known photoreceptors, it has exhibited negative phototaxis under a high intensity mercury vapor light at certain tested wavelengths from 250 to 700 nm.^61^ This response may be driven by heat, or *Tetrahymena spp.* may contain rhodopsin-like proteins involved in a light response.^62^

Beyond behavioral responses to light, which may affect zooplankton clearance rates in the dark versus light, differences in feeding approach and digestion likely influence whether zooplankton provide protection or enhancement. Both species use cilia to generate flow and direct particles toward them, but their feeding processes differ substantially. *T. pyriformis* use phagocytosis to capture larger particles and pinocytosis for smaller ones.^63–65^ While viruses have been shown in the food vacuoles of *Tetrahymena spp.*,^5,10,18^ evidence is mixed as to whether viruses are inactivated in the food vacuole.^5,10,18^ *Tetrahymena spp.* possess digestive enzymes, but the specific enzymes and their roles in viral inactivation are not known.

*B. calyciflorus* uses a ciliated corona and buccal funnel and mechanically processes food in the mastax before enzymatic digestion in the gut. For *B. calyciflorus*, viruses are likely too small to be mechanically processed, so inactivation can occur only through enzymatic digestion. *B. calyciflorus* can regulate particle ingestion and reject particles,^55^ but the viral particles may be too small to be selectively rejected. Recent work has shown that viable MS2 can be recovered from *B. plicatilis* after exposure to sunlight, suggesting that the rotifers do not digest viruses.^37^

Comparing the two organisms, *T. pyriformis* is likely better able to capture and process viruses, but their digestive residence times also differ. Full digestive cycles in *T. pyriformis* have been reported to last up to 2 h,^64^ with egestion of undigestible material sometimes occurring as early as 30 min.^64,66^ Another study showed that MS2 was inactivated within *T. thermophilia*, a closely related species to *T. pyriformis*, 24 h after coincubation.^18^ The gut passage time in *B. calyciflorus* ranges from 15 to 45 min.^58,67^ The digestive residence time could lend to physical shielding of the virus when inside the zooplankton. UV resistance of the zooplankton organisms^10^ could allow for shielding the virus from solar UV.

In addition to digestive processes impacting sunlight inactivation, zooplankton could act as exogenous photosensitizers or generate compounds that could contribute to the enhanced sunlight-mediated inactivation we observed with *T. pyriformis*. In the present experiments, ROS or sensitizers were not measured, so we cannot distinguish exogenous sunlight inactivation from the filter-feeding activity of the zooplankton. Further studies are needed to identify potential mechanisms related to digestive processes, ingestion behavior, and the production or presence of sensitizers with zooplankton.

## 4 Environmental Significance

This study demonstrates that zooplankton can either act synergistically with or impede sunlight inactivation of viruses. If zooplankton provide protection, they could act as vectors that transport infectious viruses and promote viral persistence. Conversely, if zooplankton enhances decay under sunlight, they could contribute to attenuation and be considered beneficial in lowering viral risk. In complex, multispecies communities, ecological interactions such as predation and trophic transfer can shift the balance between protection and enhanced inactivation. For example, predation of *T. pyriformis* by *B. calyciflorus* can alter exposure pathways and thereby change viral fate.^12,67,68^ Disinfection models should incorporate potential zooplankton contributions, but inclusion depends on a mechanistic understanding to predict whether zooplankton will protect viruses or promote their inactivation. Our results provide initial evidence of these contrasting roles, and targeted experiments are needed to identify biological processes and environmental drivers that determine zooplankton modulation of viral fate.

## Supplementary Material



## Data Availability

All data needed to evaluate the findings and conclusions are presented in the paper, in the Supporting Information, and in the Zenodo repository: 10.5281/zenodo.18816930.

## Notes

Text with *C. vulgaris* culture details. Text with effects of zooplankton density on viral removal. Detailed recipes for PBS (Table S1); MHSFW (Table S2); and mineral media for *C. vulgaris* (Table S3); statistical output for correlation of zooplankton densities and viral removal (Table S4); comparative analysis of virus inactivation rate constant (ratios, Table S5) and (additive, Table S6); wavelength vs Irradiance (Figure S1); experimental schematic and picture (Figure S2). *B. calyciflorus* density (Figure S3). *T. pyriformis* density (Figure S4); absorbance values for zooplankton and controls (Figure S5); references (PDF)

## References

[1] Declerck S. A. J., de Senerpont Domis L. N. (2023). Contribution
of Freshwater Metazooplankton
to Aquatic Ecosystem Services: An Overview. Hydrobiologia.

[2] Deng L., Krauss S., Feichtmayer J. (2014). Grazing of Heterotrophic
Flagellates on Viruses Is Driven by Feeding Behaviour. Environ. Microbiol. Rep..

[3] Pinheiro M. D. O., Power M. E., Butler B. J. (2007). Use of *Tetrahymena thermophila* to
Study the Role of Protozoa
in Inactivation of Viruses in Water. Appl. Environ.
Microbiol..

[4] Atanasova N. D., Dey R., Scott C. (2018). Persistence of Infectious Enterovirus within
Free-Living Amoebae–A Novel Waterborne Risk Pathway?. Water Res..

[5] Olive M., Daraspe J., Genoud C. (2023). Uptake without Inactivation
of Human Adenovirus Type 2 by *Tetrahymena Pyriformis* Ciliates. Environ. Sci.:Processes Impacts.

[6] Zhang M., Altan-Bonnet N., Shen Y. (2022). Waterborne Human Pathogenic
Viruses in Complex Microbial Communities: Environmental Implication
on Virus Infectivity, Persistence, and Disinfection. Environ. Sci. Technol..

[7] Olive M., Moerman F., Fernandez-Cassi X. (2022). Removal of Waterborne
Viruses by *Tetrahymena Pyriformis* Is
Virus-Specific and Coincides with Changes in Protist Swimming Speed. Environ. Sci. Technol..

[8] Alotaibi M. A. (2011). Internalisation
of Enteric Viruses by *Acanthamoeba castellanii*, via Ingestion of Virus-Infected Mammalian Cells. Food Environmen. Virol..

[9] Folkins M. A., Dey R., Ashbolt N. J. (2020). Interactions
between Human Reovirus and Free-Living
Amoebae: Implications for Enteric Virus Disinfection and Aquatic Persistence. Environ. Sci. Technol..

[10] Akunyili A. A., Alfatlawi M., Upadhyaya B. (2008). Ingestion without Inactivation
of Bacteriophages by *Tetrahymena*. J. Eukaryot. Microbiol..

[11] Abbas M. D., Nazir J., Stumpf P. (2012). Role of Water Fleas
(*Daphnia Magna*) in the Accumulation
of Avian Influenza Viruses from the Surrounding Water. Intervirology.

[12] Ismail N. S., Olive M., Fernandez-Cassi X. (2020). Viral Transfer and Inactivation
through Zooplankton Trophic Interactions. Environ.
Sci. Technol..

[13] Benyahya M., Laveran H., Bohatier (1997). Interactions between the Ciliated Protozoan *Tetrahymena Pyriformis* and the Simian Rotavirus SA11. Eur. J. Protistol..

[14] Frada M. J., Schatz D., Farstey V. (2014). Zooplankton May Serve
as Transmission Vectors for Viruses Infecting Algal Blooms in the
Ocean. Curr. Biol..

[15] Mayers K. M. J., Lawrence J., Skaar K. S. (2021). Removal of Large Viruses
and Their Dispersal through Fecal Pellets of the Appendicularian *Oikopleura Dioica* during *Emiliania Huxleyi* Bloom Conditions. Limnol. Oceanogr..

[16] Gerba C. P. (2007). Virus Occurrence
and Survival in the Environmental Waters. Perspect.
Med. Virol..

[17] Olive M., Gan C., Carratalà A. (2020). Control of Waterborne
Human Viruses by Indigenous Bacteria and Protists Is Influenced by
Temperature, Virus Type, and Microbial Species. Appl. Environ. Microbiol..

[18] Pinheiro M. D., Power M. E., Butler B. J. (2008). Inactivation of the
Bacteriophage MS2 by the Ciliated Protozoan, *Tetrahymena
thermophila*. Water Qual. Res.
J..

[19] Verani M., Di Giuseppe G., Tammaro C. (2016). Investigating the Role
of *Acanthamoeba polyphaga* in Protecting
Human Adenovirus from Water Disinfection Treatment. Eur. J. Protistol..

[20] Gibson K. E. (2014). Viral Pathogens
in Water: Occurrence, Public Health Impact, and Available Control
Strategies. Curr. Opin.Virol.

[21] Lanrewaju A. A., Enitan-Folami A. M., Sabiu S. (2022). Global Public Health
Implications of Human Exposure to Viral Contaminated Water. Front. Microbiol..

[22] DeFlorio-Barker S., Wing C., Jones R. M. (2018). Estimate of Incidence
and Cost of Recreational Waterborne Illness on United States Surface
Waters. Environmen. Health.

[23] Nelson K. L., Boehm A. B., Davies-Colley R. J. (2018). Sunlight-Mediated Inactivation
of Health-Relevant Microorganisms in Water: A Review of Mechanisms
and Modeling Approaches. Environ. Sci.: Processes
Impacts.

[24] Gerba C. P. (1984). Applied
and Theoretical Aspects of Virus Adsorption to Surfaces. Adv. Appl. Microbiol..

[25] Bertrand I., Schijven J. F., Sánchez G. (2012). The Impact of Temperature
on the Inactivation of Enteric Viruses in Food and Water: A Review. J. Appl. Microbiol..

[26] Moresco V., Damazo N. A., Barardi C. R. M. (2016). Thermal
and Temporal Stability on
the Enteric Viruses Infectivity in Surface Freshwater. Water Sci. Technol.: Water Supply.

[27] McMinn B. R., Rhodes E. R., Huff E. (2020). Decay of Infectious
Adenovirus and Coliphages in Freshwater Habitats Is Differentially
Affected by Ambient Sunlight and the Presence of Indigenous Protozoa
Communities. Virology Journal.

[28] Li C., Sylvestre É., Fernandez-Cassi X. (2023). Waterborne Virus Transport
and the Associated Risks in a Large Lake. Water
Res..

[29] Gerba C. P., Betancourt W. Q. (2017). Viral Aggregation:
Impact on Virus Behavior in the
Environment. Environ. Sci. Technol..

[30] Wu X., Feng Z., Yuan B. (2018). Effects of Solution
Chemistry on the Sunlight Inactivation of Particles-Associated Viruses
MS2. Colloids Surf., B.

[31] Bichai F., Payment P., Barbeau B. (2008). Protection
of Waterborne Pathogens
by Higher Organisms in Drinking Water: A Review. Can. J. Microbiol..

[32] Bichai F., Barbeau B., Payment P. (2009). Protection against UV Disinfection
of *E. Coli* Bacteria and *B.
Subtilis* Spores Ingested by *C. Elegans* Nematodes. Water Res..

[33] Bichai F., Hijnen W., Rosielle M. (2011). Preliminary Study on
the Occurrence and Risk Arising from Bacteria Internalized in Zooplankton
in Drinking Water. Water Sci. Technol..

[34] King C. H., Shotts E. B., Wooley R. E. (1988). Survival of Coliforms
and Bacterial Pathogens within Protozoa during Chlorination. Appl. Environ. Microbiol..

[35] Snelling W. J., McKenna J. P., Lecky D. M. (2005). Survival of *Campylobacter Jejuni* in Waterborne Protozoa. Appl. Environ. Microbiol..

[36] Perera I. U., Fujiyoshi S., Nishiuchi Y. (2022). Zooplankton Act as Cruise
Ships Promoting the Survival and Pathogenicity of Pathogenic Bacteria. Microbiol. Immunol..

[37] Wang J. A., Aryal O., Brownstein L. N. (2025). Zooplankton Protect
Viruses from Sunlight Disinfection. Appl. Environ.
Microbiol..

[38] Li J., Yan D., Chen L. (2019). Multiple Genotypes of Echovirus 11 Circulated
in Mainland China between 1994 and 2017. Sci.
Rep..

[39] Giammanco G. M., Filizzolo C., Pizzo M. (2024). Detection
of Echovirus
11 Lineage 1 in Wastewater Samples in Sicily. Sci. Total Environ..

[40] Maurya R., Pandey A. K. (2020). Importance of Protozoa *Tetrahymena* in Toxicological Studies: A Review. Sci. Total
Environ..

[41] Dahms H.-U., Hagiwara A., Lee J. S. (2011). Ecotoxicology, Ecophysiology, and
Mechanistic Studies with Rotifers. Aquat. Toxicol..

[42] Danes C., Cerva L. (1984). Poliovirus and Echovirus Survival in *Tetrahymena Pyriformis* Culture in Vivo. J. Hyg., Epidemiol., Microbiol.,
Immunol..

[43] US Environmental Protection Agency . Methods for Measuring the Acute Toxicity of Effluents and Receiving Waters to Freshwater and Marine Organisms US Environmental Protection Agency, Office of Water: Washington, DC; 2002.

[44] Carratalà A., Shim H., Zhong Q. (2017). Experimental Adaptation
of Human Echovirus 11 to Ultraviolet Radiation Leads to Resistance
to Disinfection and Ribavirin. Virus Evol..

[45] Pecson B.
M., Martin L. V., Kohn T. (2009). Quantitative PCR for Determining
the Infectivity of Bacteriophage MS2 upon Inactivation by Heat, UV-B
Radiation, and Singlet Oxygen: Advantages and Limitations of an Enzymatic
Treatment to Reduce False-Positive Results. Appl. Environ. Microbiol..

[46] Water Environment Federation and American Public Health Association . Standard Methods for the Examination of Water and Wastewater, 21st ed.; American Public Health Association (APHA): Washington, DC, USA, 2005.

[47] Mattle M. J., Vione D., Kohn T. (2015). Conceptual Model and
Experimental
Framework to Determine the Contributions of Direct and Indirect Photoreactions
to the Solar Disinfection of MS2, phiX174, and Adenovirus. Environ. Sci. Technol..

[48] Wang Y., He G. X., Sanchez-Quete F. (2025). Systematic Review and
Meta-Analysis on the Inactivation Rate of Viruses and Bacteriophage
by Solar Wavelength Radiation. Environ. Sci.
Technol..

[49] Fisher M. B., Love D. C., Schuech R. (2011). Simulated Sunlight Action
Spectra for Inactivation of MS2 and PRD1 Bacteriophages in Clear Water. Environ. Sci. Technol..

[50] Sharpless C. M., Blough N. V. (2014). The Importance of
Charge-Transfer Interactions in Determining
Chromophoric Dissolved Organic Matter (CDOM) Optical and Photochemical
Properties. Environ. Sci.: Processes Impacts.

[51] Kohn T., Nelson K. L. (2007). Sunlight-Mediated
Inactivation of MS2 Coliphage via
Exogenous Singlet Oxygen Produced by Sensitizers in Natural Waters. Environ. Sci. Technol..

[52] Meister S., Verbyla M. E. (2018). Variability
in Disinfection Resistance between
Currently Circulating *Enterovirus B* Serotypes and
Strains. Environ. Sci. Technol..

[53] Silverman A. I., Peterson B. M., Boehm A. B. (2013). Sunlight Inactivation
of Human Viruses and Bacteriophages in Coastal Waters Containing Natural
Photosensitizers. Environ. Sci. Technol..

[54] Starkweather P. L., Gilbert J. J., Frost T. M. (1979). Bacterial Feeding by the Rotifer *Brachionus
Calyciflorus*: Clearance and Ingestion
Rates, Behavior and Population Dynamics. Oecologia.

[55] Gilbert J.
J., Starkweather P. L. (1977). Feeding
in the Rotifer *Brachionus Calyciflorus*: I. Regulatory Mechanisms. Oecologia.

[56] Lavin D. P., Hatzis C., Rienc F. (1990). Size Effects on the
Uptake of Particles by Populations of *Tetrahymena Pyriformis* Cells. J. Protozool..

[57] Bischel H. N., Schertenleib A. (2015). Inactivation Kinetics and Mechanisms of Viral
and Bacterial Pathogen Surrogates during Urine Nitrification. Environ. Sci.: Water Res. Technol..

[58] Starkweather, P. L. Aspects of the Feeding Behavior and Trophic Ecology of Suspension-Feeding Rotifers. In Rotatoria; Dumont, H. J. ; Green, J. , Eds.; Springer Netherlands: Dordrecht, 1980; pp 63–72 10.1007/978-94-009-9209-2_13.

[59] Kim H.-J., Lee J.-S., Hagiwara A. (2018). Phototactic Behavior of Live Food
Rotifer *Brachionus Plicatilis* Species
Complex and Its Significance in Larviculture: A Review. Aquaculture.

[60] Colangeli P., Schlägel U. E., Obertegger U. (2019). Negative Phototactic
Response to UVR in Three Cosmopolitan Rotifers: A Video Analysis Approach. Hydrobiologia.

[61] Kim D. H., Casale D., Kőhidai L. (2009). Galvanotactic and Phototactic
Control of *Tetrahymena Pyriformis* as
a Microfluidic Workhorse. Appl. Phys. Lett..

[62] Rainville, J. A Search for Light-Detecting Proteins in the Free-Living Protist, *Tetrahymena thermophila*: Does *Tetrahymena* Have Opsin-like or Bacteriorhodopsin-like Proteins? Senior Honors Projects 2013.

[63] Nilsson J. R. (1977). Fine Structure
and RNA Synthesis of *Tetrahymena* during Cytochalasin
B Inhibition of Phagocytosis. J. Cell Sci..

[64] Nilsson J. R. (1977). On Food
Vacuoles in *Tetrahymena Pyriformis* GL. J. Protozool..

[65] Nilsson J. R., Deurs B. V. (1983). Coated Pits and
Pinocytosis in *Tetrahymena*. J. Cell Sci..

[66] Blum J. J., Greenside H. (1976). Particle Ejection from the Cytoproct of *Tetrahymena*. J. Protozool..

[67] Joaquim-Justo C., Detry C., Caufman F. (2004). Feeding of Planktonic
Rotifers on Ciliates: A Method Using Natural Ciliate Assemblages Labelled
with Fluorescent Microparticles. J. Plankton
Res..

[68] Mohr S., Adrian R. (2000). Functional Responses of the Rotifers *Brachionus Calyciflorus* and *Brachionus Rubens* Feeding on Armored and Unarmored Ciliates. Limnol. Oceanogr..

