# Supplementary material for: Differential Effects of Zooplankton on Sunlight Inactivation of Viruses
Source: Environ Sci Technol. 2026 May 26;60(22):16204–12. doi: 10.1021/acs.est.6c03486 (PMC13262028; doi:10.1021/acs.est.6c03486)
Supplement: Supplementary file 1 [file es6c03486_si_001.pdf]

Supporting Information

Differential Effects of Zooplankton on Sunlight Inactivation of Viruses

Martha I. Verbel-Olarte[a], Tamar Kohn[a], Niveen S. Ismail[b,*]

[a] Laboratory of Environmental Virology, School of Architecture, Civil and Environmental Engineering (ENAC), École Polytechnique Fédérale de Lausanne (EPFL), 1015 Lausanne, Switzerland

[b] Picker Engineering Program, Smith College, Northampton, Massachusetts 01063, United States.

* Corresponding author:

nismail@smith.edu (Niveen S. Ismail)

Number of Pages: 14

SI Text: 2 pages

Number of Tables: 6

Number of Figures: 5

SI References: 1 page

Chlorella vulgaris Culture and Preparation

The freshwater microalgae *Chlorella vulgaris,* which were used as the primary food source for *B. calyciflorus*, were kindly provided by the Ludwig Group at EPFL. Individual colonies were selected from the agar plate stocks and batch-cultured in 100 mL of sterile mineral medium (Table S3) [1–3] in 250 mL Erlenmeyer flasks with cotton wool plugs. The Erlenmeyer flasks were shaken at 130 rpm on a platform shaker (C10, New Brunswick Scientific) and illuminated from below at 100 ± 5 µmol photons m^{-2} s^{-1}on an 18:6 hour light: dark cycle.

Effects of zooplankton density variability on viral removal

Zooplankton densities were of a similar order of magnitude to values reported in prior virus and zooplankton co-incubation and clearance-rate studies[4–6]. *Tetrahymena pyriformis* densities were consistent across experiments and among beakers (Figure S4). The *B. calyciflorus* suspensions were relatively dilute compared with the *T. pyriformis* suspensions, and initial rotifer densities varied between beakers, which is expected when sampling live, fast-swimming rotifers in a dilute suspension (Figure S3). For example, 6 beakers prepared from a single rotifer culture and sampled sequentially in triplicate had densities ranging from 122 to 178 rotifers $mL^{-1,}$ with a mean standard deviation of approximately 30 rotifers mL^{-1}. For both *T. pyriformis* and *B. calyciflorus*, we confirmed that there was no significant correlation between zooplankton density and net viral log removal for MS2 and E11 (Spearman's, $p>0.05$, Table S4).

Table S1. Phosphate buffer saline (PBS) using Milli-Q Water

**Reagents**	**Chemical formula**	**Concentration [g L^{-1}]**	**Producer**
disodium hydrogen phosphate	Na_2HPO_4	0.71	Acros organics
sodium chloride	NaCl	0.58	Acros organics

Solution was autoclaved to sterilize and was adjusted to pH 7.4

Table S2. Moderately hard synthetic freshwater (MHSFW) using Milli-Q Water

**Reagents**	**Chemical formula**	**Concentration [mg L^{-1}]**	**Producer**
sodium bicarbonate	$NaHCO_3$	96	Acros organics
calcium sulfate dihydrate*	$CaSO_4 \bullet 2H_2O$	60	AppliChem
magnesium sulfate heptahydrate	$MgSO_4 \bullet 7H_2O$	122.865	AppliChem
potassium chloride	KCl	4	AppliChem

*This reagent was mixed separately for 24 h on a magnetic stirrer.

After mixing all the chemicals, the MHSFW was aerated vigorously for 48 h

Table S3: Mineral medium preparation for *Chlorella vulgaris* growth using Milli-Q water

Reagents	Chemical formula	Concentration [mg L^{-1}]	Producer
sodium nitrate	$NaNO_3$	1557	Sigma-Aldrich
monopotassium phosphate	KH_2PO_4	118.5	Sigma-Aldrich
magnesium sulfate heptahydrate	$MgSO_4 \bullet 7H_2O$	102	Sigma-Aldrich
	EDTA-FeNa	20	Sigma-Aldrich
calcium chloride hexahydrate	$CaCl_2 \bullet 6H_2O$	86.8	Sigma-Aldrich
boric acid	H_3BO_3	0.415	Sigma-Aldrich
copper (II) sulfate pentahydrate	$CuSO_4 \bullet 5H_2O$	0.475	Sigma-Aldrich
manganese (II) chloride tetrahydrate	$MnCl_2 \bullet 4H_2O$	1.65	Sigma-Aldrich
cobalt(II) sulfate heptahydrate	$CoSO_4 \bullet 7H_2O$	0.3	Sigma-Aldrich
zinc sulfate heptahydrate	$ZnSO_4 \bullet 7H_2O$	1.35	Sigma-Aldrich
ammonium heptamolybdate tetrahydrate	$(NH_4)_6 Mo_7 O_{24} \bullet 4H_2O$	0.085	Sigma-Aldrich
ammonium metavanadate	NH_4VO_3	0.007	Sigma-Aldrich

The pH was adjusted to 7.00 ± 0.05.

Table S4. Statistical output (Spearman's Rank Order) for zooplankton density correlated with the log removal of viruses

Experiment Type	Zooplankton	Virus	Spearman's Rho	Significance (p-value)
Dark	*B. calyciflorus*	MS2	-0.486	0.329
		E11	0.371	0.468
Light		MS2	-0.200	0.704
		E11	0.290	0.577
Dark	*T. pyriformis*	MS2	-0.600	0.208
		E11	0.667	0.148
Light		MS2	0.257	0.623
		E11	-0.257	0.623

Table S5. Virus inactivation rate constant ratios based on time (k, hr^{-1}) and fluence (κ, m^2 kJ^{-1}) under light and dark conditions

**k or *κ*- value Ratio (Experimental/Control)**	***T pyriformis***	***B. calyciflorus***
Dark E11 ($k_{exp}/k_{control}$)	1.8	3.8
Light E11 ($\kappa_{exp}/\kappa_{control}$)	3.1	0.57
Dark MS2 ($k_{exp}/k_{control}$)	0.66	2.3
Light MS2 ($\kappa_{exp}/\kappa_{control}$)	3.2	1.3

Table S6. Additive and observed light inactivation rate constants (k, hr^{-1})

Virus	$k_{\text{Dark, }\textit{T. pyriformis}} + k_{\text{Light, virus-only control}}$	$k_{\text{light, }\textit{T. pyriformis}}$
MS2	0.11	0.15
E11	0.30	0.37

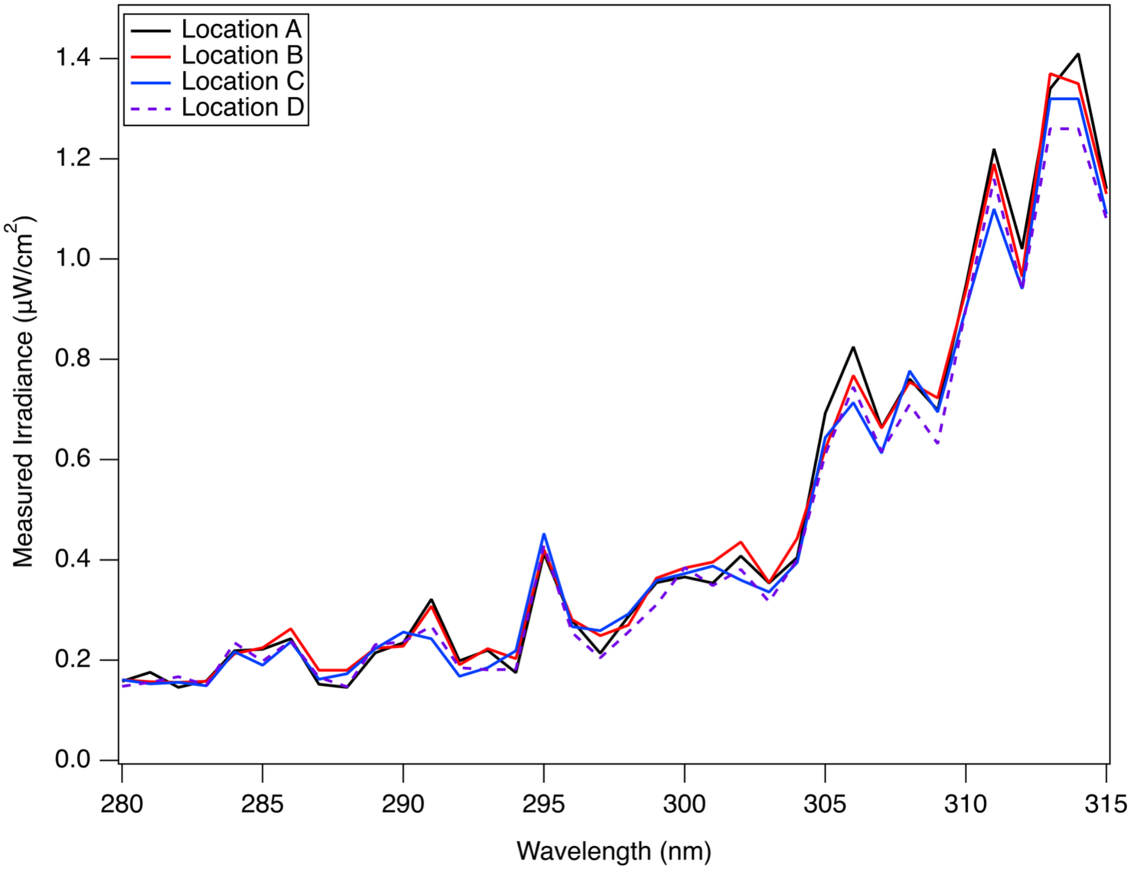

Figure S1. Irradiance of the 4 locations of beakers under the solar simulator for the UVB wavelength range (280-315 nm).

A

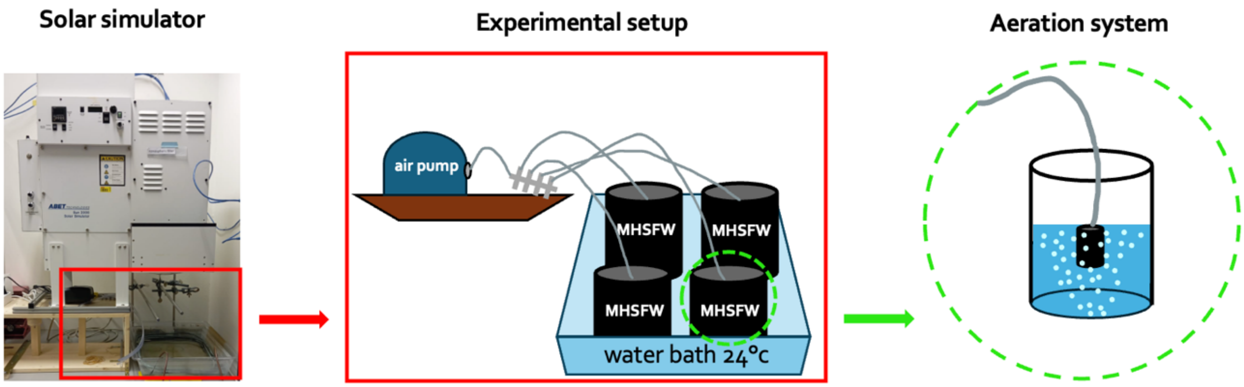

B

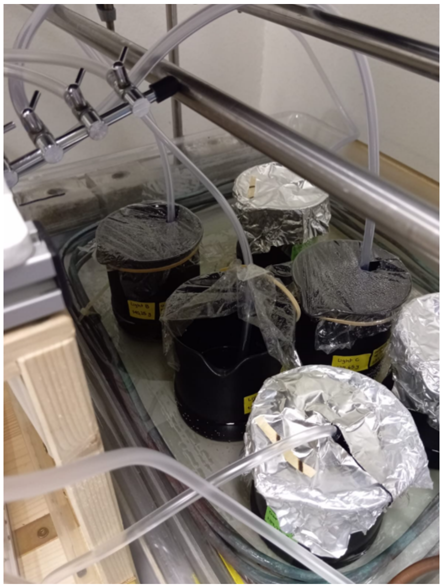

Figure S2. A) Schematic of the experimental setup for the light experiments. B) Example of light and dark experiment beakers. Beakers were covered with plastic film, and dark experiment beakers were additionally covered with foil.

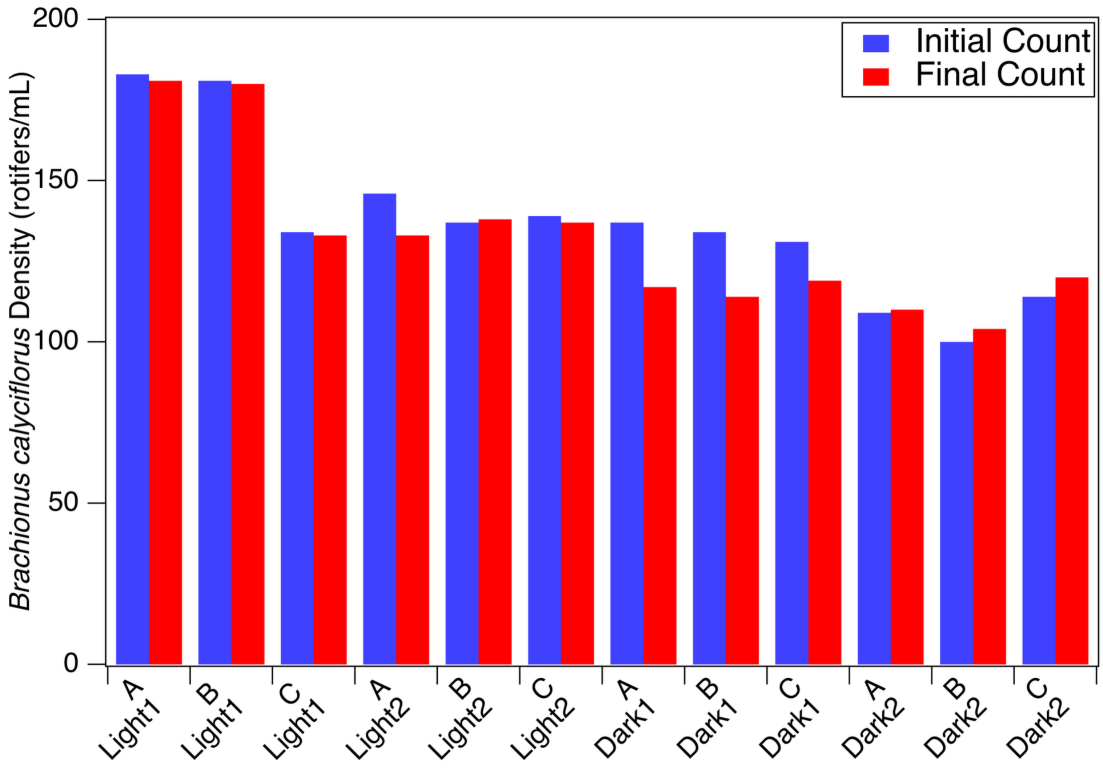

Figure S3. *Brachionus calyciflorus* density at the start and completion of dark and light experiments. The letters A, B, and C represent experimental replicates for each experiment type and correspond to the location beneath the solar simulator. Light represents sunlight exposure experiments, with the numbers 1 and 2 representing the first and second experiments. Dark represents the dark experiments, with numbers 1 and 2 representing the first and second experiments.

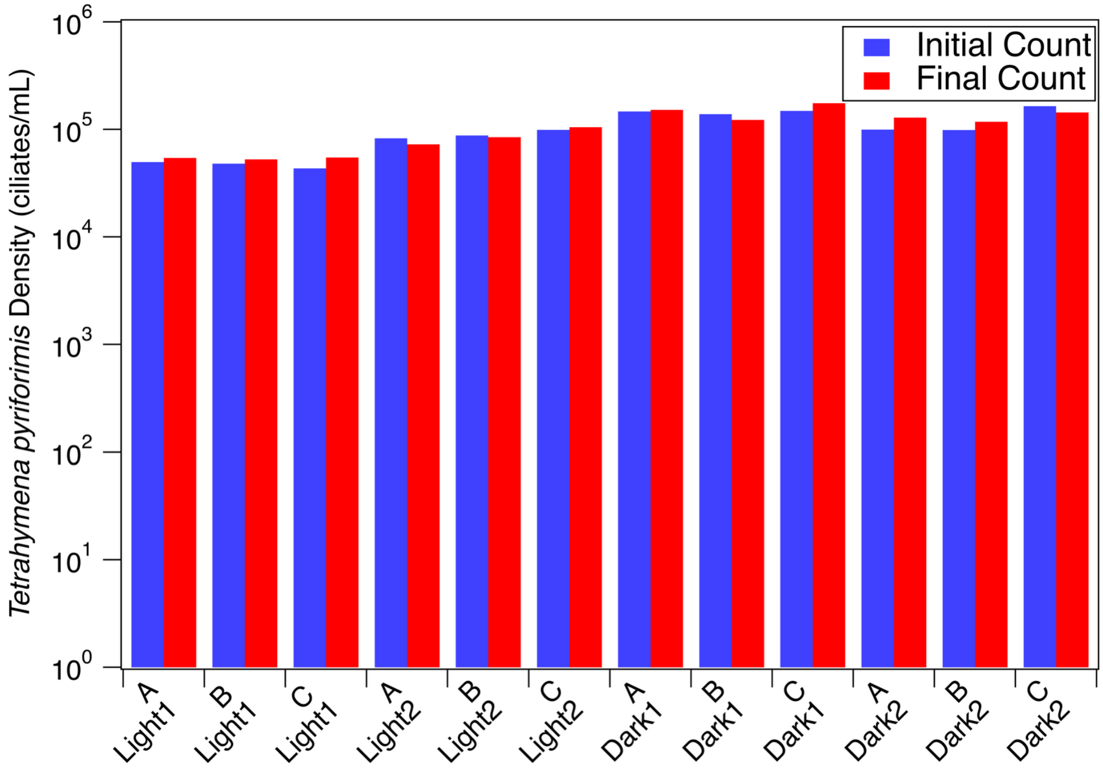

Figure S4. *Tetrahymena pyriformis* density at the start and completion of each experiment. The letters A, B, and C represent experimental replicates for each experiment type. Light represents sunlight exposure experiments, with the numbers 1 and 2 representing the first and second experiments. Dark represents the dark experiments, with numbers 1 and 2 representing the first and second experiments.

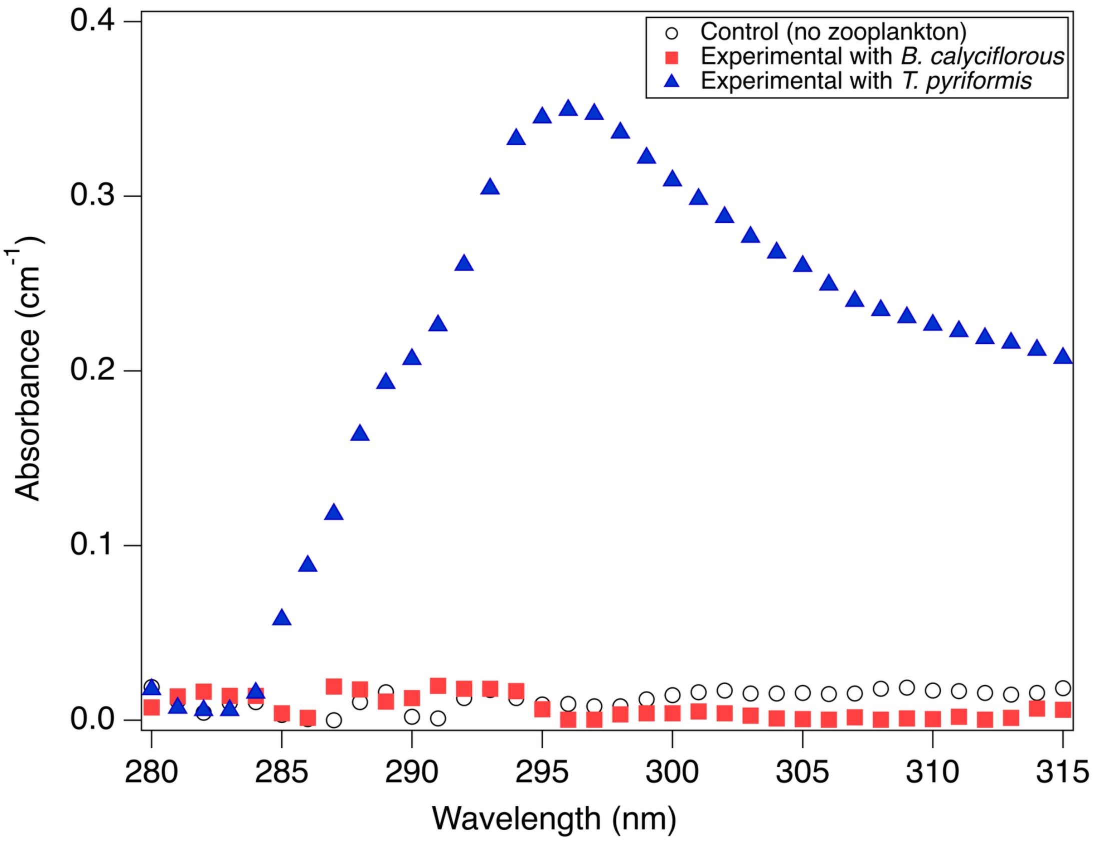

Figure S5. Average absorbance values for moderately hard synthetic freshwater with *T. pyriformis*, *B. calyciflorous,* and the control without zooplankton

REFERENCES

(1) Pulgarin, A.; Giannakis, S.; et al. A Novel Proposition for a Citrate-Modified Photo-Fenton Process against Bacterial Contamination of Microalgae Cultures. *Applied Catalysis B: Environmental* **2020**, *265* (October 2019), 118615. https://doi.org/10.1016/j.apcatb.2020.118615.

(2) Zanchetta, E.; Ollivier, M.; et al. Abiotic Stress Approaches for Enhancing Cellulose and Chitin Production in *Chlorella Vulgaris*. *International Journal of Biological Macromolecules* **2025**, *309*, 142969.

(3) Doušková, I.; Kaštánek, F.; et al. Utilization of Distillery Stillage for Energy Generation and Concurrent Production of Valuable Microalgal Biomass in the Sequence: Biogas-Cogeneration-Microalgae-Products. *Energy Conversion and Management* **2010**, *51* (3), 606–611. https://doi.org/10.1016/j.enconman.2009.11.008.

(4) Olive, M.; Moerman, F.; et al. Removal of Waterborne Viruses by *Tetrahymena Pyriformis* Is Virus-Specific and Coincides with Changes in Protist Swimming Speed. *Environmental Science & Technology* **2022**, *56* (7), 4062–4070.

(5) Wang, J. A.; Aryal, O.; et al. Zooplankton Protect Viruses from Sunlight Disinfection. *Appl Environ Microbiol* **2025**, *91* (4). https://doi.org/10.1128/aem.02540-24.

(6) Ismail, N. S.; Blokker, B. M.; et al. Impact of Metazooplankton Filter Feeding on *Escherichia Coli* under Variable Environmental Conditions. *Appl.Environ.Microbiol.* **2019**, *85* (23).
